# Effects of Sludge Retention Times on Nutrient Removal and Nitrous Oxide Emission in Biological Nutrient Removal Processes

**DOI:** 10.3390/ijerph110403553

**Published:** 2014-03-27

**Authors:** Bo Li, Guangxue Wu

**Affiliations:** Key Laboratory of Microorganism Application and Risk Control (MARC) of Shenzhen, Graduate School at Shenzhen, Tsinghua University, Shenzhen 518055, Guangdong, China; E-Mail: libotsinghuasz@163.com

**Keywords:** sludge retention time, nitrous oxide, endogenous decay rate, greenhouse gas, excess sludge production

## Abstract

Sludge retention time (SRT) is an important factor affecting not only the performance of the nutrient removal and sludge characteristics, but also the production of secondary pollutants such as nitrous oxide (N_2_O) in biological nutrient removal (BNR) processes. Four laboratory-scale sequencing batch reactors (SBRs), namely, SBR5, SBR10, SBR20 and SBR40 with the SRT of 5 d, 10 d, 20 d and 40 d, respectively, were operated to examine effects of SRT on nutrient removal, activated sludge characteristics and N_2_O emissions. The removal of chemical oxygen demand or total phosphorus was similar under SRTs of 5–40 d, SRT mainly affected the nitrogen removal and the optimal SRT for BNR was 20 d. The molecular weight distribution of the effluent organic matters was in the range of 500–3,000 Da under SRTs of 5–40 d. The lowest concentration of the effluent soluble microbial products concentration was obtained at the SRT of 5 d. Nitrifier growth was limited at a short SRT and nitrite existed in the effluent of SBR5. With increasing SRTs, mixed liquor suspended solids concentration increased while the excess sludge production was reduced due to the high endogenous decay rate at high SRTs. Endogenous decay coefficients were 0.020 d^−1^, 0.036 d^−1^, 0.037 d^−1^ and 0.039 d^−1^ under SRTs of 5–40 d, respectively. In BNR, the N_2_O emission occurred mainly during the aerobic phase and its emission ratio decreased with increasing SRTs. The ratio between the N_2_O-N emission and the removed ammonium nitrogen in the aerobic phase was 5%, 3%, 1.8% and 0.8% at the SRT of 5 d, 10 d, 20 d and 40 d, respectively. With low concentrations of dissolved oxygen and high concentrations of oxidized nitrogen, the N_2_O emission was significantly accelerated due to heterotrophic denitrification activities.

## 1. Introduction

The increasing occurrence of eutrophication has become a serious environmental problem. Nutrients such as nitrogen and phosphorus have been recognized as the major factors inducing eutrophication. The removal of nutrients, especially nitrogen and phosphorus, has become the primary object of wastewater treatments. Biological nutrient removal (BNR) processes have been widely used in wastewater treatment and activated sludge process is the dominant BNR process. Typically, BNR includes anaerobic, anoxic and aerobic phases for nitrification, denitrification and phosphorus removal, respectively. Sludge retention time (SRT) is an important factor which affects not only the performance of nutrient removal and sludge characteristics but also the production of secondary pollutants such as nitrous oxide (N_2_O) in the BNR. Nowadays, lots of wastewater treatment plants are operated at long SRTs both to enhance nitrification and reduce excess sludge production. Therefore, it is important to examine the effect of SRT on the BNR.

Ammonium nitrogen (NH_4_-N) can be removed by aerobic nitrification followed by anoxic denitrification. As autotrophic nitrifiers grow very slowly, a long SRT is needed to maintain a certain amount of nitrifiers and ensure effective nitrification. Enhanced biological phosphorus removal (EBPR) is achieved by the discharge of phosphorus enriched sludge. However, a long SRT may decrease the efficiency of phosphorus removal due to the low sludge wasting rate and the possible phosphorus release in the clarifier. Pai *et al.* [1] reported that with the SRT reduced from 15 d to 5 d, the removal efficiency of NH_4_-N decreased from 90% to 26%, and the removal efficiency of total nitrogen (TN) decreased from 14% to 8%. Kargi and Uygur [2] investigated BNR in sequencing batch reactors (SBRs) at SRTs of 5–30 d and results showed that simultaneous nitrogen and phosphorus removal could be achieved effectively under SRTs of 10 and 15 d, but the NH_4_-N removal efficiency decreased with the SRT above 15 d due to high endogenous decay of microorganisms at high SRTs. There are different opinions on the effect of high SRT on the phosphorus removal. Some researchers reported that the phosphorus removal efficiency decreased with increasing SRTs due to the decreased biomass yield rate, while others showed that polyphosphate accumulating organisms (PAOs) would be dominant in the systems and the phosphorus removal efficiency increased under a long SRT due to the lower decay rate of PAOs than that of other microorganisms [3,4]. Lee *et al.* [5] showed that although the sludge discharging rate decreased with increasing SRTs, a high amount of PAO_S_ in the system and a high phosphorus content inside PAOs occurred, the amount of PAOs and the phosphorus wasting load should be balanced to improve the phosphorus removal efficiency. During BNR, soluble microbial products (SMP), mainly composed of carbohydrate, protein and humic substances, are produced. The presence of SMP may not only affect the effluent quality of wastewater treatment but also form toxic byproducts during the chlorination [6,7]. As SRT affects the metabolism of microorganisms, it also affects the SMP formation. Some researchers found that the concentration of SMP decreased with increasing SRTs and SMP could be better removed at high SRTs [8,9].

A large amount of waste sludge may be produced during BNR. The cost of sludge disposal is extremely high and may account for up to 60% of the operating cost in a wastewater treatment plant (WWTP) [10]. Many WWTPs tend to operate treatment systems under a high SRT to enhance endogenous respiration and to reduce excess sludge production. SRT affects not only sludge production but also sludge activity. With a prolonged SRT, although the concentration of activated sludge increases, the nutrient removal performance may decrease due to the reduced sludge activity. Therefore, it is meaningful to investigate the sludge characteristics under different SRTs such as sludge yield coefficient, sludge activity and so on to determine the optimal SRT. Huang *et al.* [11] showed that activities of both organic decomposition and nitrification decreased with SRTs above 10 d. In addition, SRT also affects the physical characteristics such as the sludge settling characteristics. Extracellular polymeric substances (EPS) play an important role in the sludge settling, and their production is also affected by SRT [12,13]. Liao *et al.* [13] considered that the EPS content was independent with SRT while Al-Halbouni *et al.* [14] and Cho *et al.* [15] showed that more EPS were produced at short SRTs. In addition, there are different opinions on the effect of SRT on the sludge settling. Some showed that the absence of EPS might prevent the formation of large size flocs and lead to poor settling [12,13,14], while others considered that some EPS components might enhance the sludge settling [16]. Shin *et al.* [17] argued that sludge settling had no relationship with the amount of EPS but a high ratio of carbohydrate to protein tended to cause poor sludge settling.

Along with BNR, production of secondary pollutants such as N_2_O has been received much attention and become a hot research issue [18]. N_2_O is mainly produced during biological nitrogen removal including both nitrification and denitrification, and further clarification of the N_2_O emission mechanism is still required [19,20]. There are three microbial pathways involved in the production of N_2_O: (i) during nitrification, from hydroxylamine (NH_2_OH) oxidation [21]; (ii) during denitrification, as an intermediate during reduction of nitrate to N_2_ by heterotrophic denitrifiers [22]; and (iii) during nitrification, from nitrifier denitrification using nitrite as the electron acceptor [23,24]. Generally, a high N_2_O emission occurs at low SRTs. Noda *et al.* [25] compared the performance of SBRs under SRTs of 5 d, 10 d and 20 d, and found that with decreasing SRTs, the N_2_O emission increased while the nitrification efficiency decreased. Shan *et al.* [26] examined the N_2_O emission of BNR under SRTs of 9 d and 15 d, and showed that most of the N_2_O emission came from the aerobic phase and there was more N_2_O emission at low SRTs, although high nitrification efficiency was achieved in both systems. However, previous studies do not explain why more N_2_O is produced under low SRTs.

In this study, four lab-scale BNR process were operated to examine the operating performance at different SRTs, including nutrient removal, sludge characteristics and the N_2_O emission, with the aim of providing valuable information for operation of WWTPs.

## 2. Materials and Methods

### 2.1. Wastewater Treatment Systems and Their Operation

Four 6-litre lab-scale SBRs, namely, SBR5, SBR10, SBR20 and SBR40, were operated at 25 °C for biological nutrient removal. The SBRs were operated four cycles per day and each cycle comprised the following phases: fill/anoxic (10 min), anoxic/anaerobic (110 min), aerobic (180 min), settlement (40 min) and draw/idle (20 min). In each SBR cycle, 2 L of treated wastewater were exchanged with a new batch of synthetic wastewater. The reactor was well mixed during the fill and anaerobic/anoxic phase. During the aerobic phase, air was supplied with an air diffuser located at the bottom of the reactor. The dissolved oxygen (DO) concentration was above 3 mg/L during the aerobic phase. The SRT of each SBR was controlled by wasting activated sludge at the end of the aerobic phase and SRTs were controlled at 5 d, 10 d, 20 d and 40 d, respectively.

All SBRs were fed with synthetic wastewater composed of 200 mg/L sodium acetate, 280 mg/L sucrose, 10 mg/L yeast extract, 153 mg/L NH_4_Cl, 200 mg/L NaHCO_3_, 37 mg/L Na_2_HPO_4_, 90 mg/L MgSO_4_·7H_2_O, 14 mg/L CaCl_2_·6H_2_O, and 0.4 mL/L of trace elements. The component of trace elements was following Smolders *et al.* [27]. The reactors were seeded with activated sludge taken from a wastewater treatment plant in Shenzhen, China.

The performance of nutrient removal was investigated by examination of the influent and effluent parameters and dynamics of nutrients in typical SBR cycles. The influent chemical oxygen demand (COD), NH_4_-N and orthophosphate (PO_4_-P) and the effluent COD, NH_4_-N, nitrite nitrogen (NO_2_-N), nitrate nitrogen (NO_3_-N), PO_4_-P, TN, total phosphorus (TP) and suspended solids (SS) were determined. Total organic carbon (TOC), protein and molecular weight distribution (MWD) of organic matters in the effluent were also determined. The ratio of ultraviolet absorbance at 254 nm (UVA_254_) and TOC was used as a surrogate parameter to determine the humic content [28,29]. SS, volatile suspended solids (VSS), sludge volume index (SVI) and EPS were analyzed for solid samples.

### 2.2. Sludge Activity at Different SRTs

Sludge activity experiments were conducted after the reactors reached steady state. To test the nitrification rate (including the oxidation of NH_4_-N and NO_2_-N) and the denitrification rate (including the reduction of NO_3_-N and NO_2_-N), activated sludge mixed liquor was withdrawn from the SBR reactors at the end of the aerobic phase and used in the batch experiments. The batch reactors were made from 500 mL capped glass flasks. The supernatant of the mixed liquor was replaced with synthetic wastewater which had the same components as the influent but without nitrogen and carbon sources. The temperature of batch reactors was maintained at 25 °C.

When testing the oxidation rate of NH_4_-N or NO_2_-N, NH_4_-N or NO_2_-N was added to achieve an initial concentration of 20 mg/L NH_4_-N or 10 mg/L NO_2_-N, and air was supplied during the whole nitrification period. Samples were taken and the pH and DO in reactors were tested every 10 min. Samples were then centrifuged to analyze dynamics of soluble nitrogen parameters.

During testing the reduction rate of NO_3_-N or NO_2_-N, carbon source and NO_3_-N or NO_2_-N were added into the batch reactors to achieve initial concentrations of 400 mg/L COD and 30 mg/L NO_3_-N or 20 mg/L NO_2_-N. Samples were taken every 10 min and then centrifuged to analyze dynamics of soluble nitrogen parameters.

### 2.3. N_2_O Emission at Different SRTs

To examine N_2_O emission during SBR cycles at different SRTs, 2 L of activated sludge mixed liquor were withdrawn from each SBR at the end of the aerobic phase and transferred to a batch SBR reactor made from a 2 L capped glass flask, each with three ports on the cap, one for aeration, one for liquid sampling and the last one for gas sampling and measurement of the air flow rate. 1/3 of the supernatant of the mixed liquor was replaced with the influent synthetic wastewater. Then the batch reactors experienced a SBR cycle comprising the anoxic/anaerobic phase (2 h) and the aerobic phase (3 h). Magnetic stirrers were used during the anoxic/anaerobic phase and aeration was provided during the aerobic phase. Water and gas samples were taken at intervals to test NH_4_-N, NO_2_-N, NO_3_-N and N_2_O (in gas). DO and pH in reactors were also measured. N_2_ was supplied during the anoxic phase after sampling for the balance of gas pressure.

As results of cycle experiments revealed that most of the N_2_O emissions occurred during the aerobic phase, experiments were further conducted to investigate the N_2_O emission mechanism during the aerobic phase. Three L of activated sludge mixed liquor were withdrawn from each SBR reactor at the end of the anaerobic phase and divided equally into three batch reactors made from 1 L capped glass flasks, each with three ports on the cap, one for liquid sampling, one for aeration and the other for gas sampling and measurement of the air flow rate. Magnetic stirrers were used to stir the liquor. Three experiment conditions were examined in this experiment: (i) NH_4_-N was the only nitrogen component, and no allyl thiourea (ATU) or oxidized nitrogen was added; (ii) ATU was added to inhibit nitrification, and NO_3_-N and NO_2_-N were added to achieve the initial concentration of 20 mg/L and 10 mg/L. As nitrification was inhibit, all the N_2_O emission was due to denitrification during the aerobic phase; (iii) No oxidized nitrogen but ATU was added, neither nitrification nor denitrification would take place, and the N_2_O emission under this condition was used as the control. By comparing results from (i) and (ii), the N_2_O emission mechanism would be clarified further.

### 2.4. Analytical Methods

All the collected water samples, except samples for the TN and TP tests, were centrifuged at 10,000× *g* (L-550, Anting, Shanghai, China) for 2 min or filtered through 0.45 μm acetate fiber membrane filters (Millipore Swinnex-25, Billerica, MA, USA). COD, NH_4_-N, NO_2_-N, NO_3_-N, PO_4_-P, TN, TP, SS, VSS and SVI were determined according to standard methods [30] The pH and DO were measured using probes of WTW pH 3110 and WTW Oxi315i (WTW, Munich, Germany), respectively.

EPS were extracted using the formaldehyde plus NaOH method [31]. After extraction, the mixed liquor was centrifuged at 10,000× *g* for 2 min for the analysis of protein and carbohydrate. The protein was analyzed by the Lowry method [32], and bovine serum albumin was used as the standard. The carbohydrate was analyzed by the phenol-concentrated sulfuric acid method [33], and glucose was used as the standard.

TOC was determined by a TOC analyzer (Shimadzu, Kyoto, Japan). UVA_254_ was analyzed by a UV-1800 PC spectrophotometer (Aoyi, Shanghai, China) at a wavelength of 254 nm using a 1 cm quartz cell. The MWD of organic matters in the effluent was measured using a high-pressure size exclusion chromatography (HPSEC) system (LC-20AD, Shimadzu) and salicylic acid was used as the standard according to the method developed by Zhou *et al.* [34].

N_2_O was detected by a gas chromatography (GC, Agilent 6820, Agilent Technologies, Wilmington, DE, USA) with an electron capture detector and a HP-PLOT/Q column (J&W GC Columns, Agilent Technologies). Temperatures during testing were 50 °C for the injector, 50 °C for the oven, and 300 °C for the detector. Nitrogen gas was used as the carrier gas at the flow rate of 15 mL/min. Pure N_2_O gas was used as the standard for calibration. For convenient comparison, the produced N_2_O in the gas phase was expressed as mg/L, representing mg N_2_O (gas) produced from the specific volume (L) of mixed activated sludge liquor. The N_2_O emission rate was calculated according to Noda *et al.* [25].

## 3. Results and Discussion

### 3.1. Performance of Nutrient Removal at Different SRTs

Performance of nutrient removal in SBRs at different SRTs under steady state is summarized in Table 1.

**Table 1:** Parameters of the influent and effluent of all SBRs at different SRTs.

Parameter		Unit	Value			
SRT		d	5	10	20	40
COD_in_		mg/L	399.3 ± 20.6			
NH_4_-N_in_		mg/L	40.6 ± 1.4			
PO_4_-P_in_		mg/L	8.4 ± 0.8			
COD_eff_		mg/L	20.1 ± 3.4	17.5 ± 3.5	13.6 ± 2.6	18.5 ± 4.7
TN_eff_		mg/L	9.2 ± 1.8	11.3 ± 1.6	11.2 ± 1.7	11.7 ± 0.6
TP_eff_		mg/L	3.6 ± 1.5	3.7 ± 0.6	3.7 ± 1.1	3.1 ± 0.9
NH_4_-N_eff_		mg/L	0.2 ± 0.1	0.1 ± 0.1	0.1 ± 0.1	0
NO_3_-N_eff_		mg/L	5.9 ± 0.9	6.7 ± 1.6	6.8 ± 0.7	8.9 ± 0.9
NO_2_-N_eff_		mg/L	2.6 ± 1	0	0	0
PO_4_-P_eff_		mg/L	2.0 ± 0.8	2.6 ± 1.3	1.9 ± 0.8	1.8 ± 0.5
SS		mg/L	20.3 ± 4.3	19.3 ± 6.7	17.6 ± 5.4	19.1 ± 4.9
SMP	TOC	mg/L	7.8 ± 0.6	13.3 ± 1.8	12 ± 3.7	9 ± 1.7
	Protein	mg/L	5.44	6.44	4.19	4.19
	UVA_254_	1/cm	0.08	0.07	0.07	0.06

Average COD removal percentages of four SBRs were 95%, 96%, 97% and 95%, illustrating that all SBRs had good capacity of COD removal and the COD removal was not affected by SRT. This could be due to that heterotrophs had a fast growth rate and had privilege in the competition with other microorganisms. Phosphorus removal efficiencies varied slightly with SRTs and ranged between 56%–58%.

All SBRs had good NH_4_-N removal performance with removal percentages above 99%. The high removal efficiency of NH_4_-N might be due to the fact that SRTs were long enough and oxygen supply was sufficient for the growth of nitrifiers. NO_2_-N existed in the effluent of SBR5 showed that partial nitrification occurred, which could be due to the high lysis rate of NOB at low SRTs. Pai *et al.* [1] obtained that at the SRT of 5 d, the lysis rate was 0.13 d^−1^ for AOB and 0.18 d^−1^ for NOB. Pollice *et al.* [35] showed that when DO was not a limiting factor, SRT was the key factor determining partial nitrification and partial nitrification was easily achieved at a short SRT. Therefore, SRTs above 10 d should be maintained in a BNR process to ensure complete nitrification.

The effluent oxidized nitrogen in each SBR was 8.5 mg/L, 6.7 mg/L, 6.8 mg/L and 8.9 mg/L, respectively. As oxidized nitrogen was mainly removed through denitrification in the anoxic phase, an optimal SRT existed for denitrificaton. At the SRT of 5 d, there was less denitrifiers in the system while at the SRT of 40 d, the denitrification might decrease due to the high decay rate of denitrifiers. Tremblay *et al.* [36] reported that the concentration of the oxidized nitrogen was low under SRTs between 10–15 d and increased until the SRT of 30 d. A proper SRT should be maintained in a BNR system to achieve effective denitrification.

**Figure 1:**
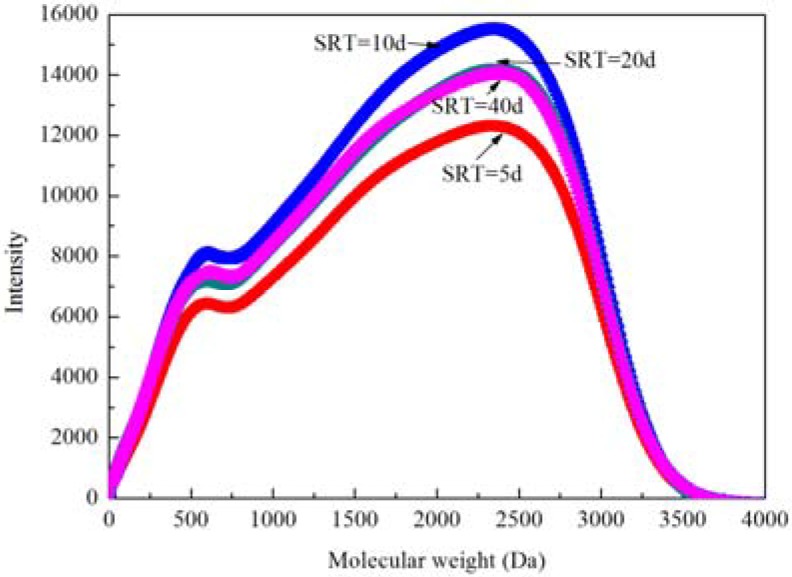
The molecular weight distribution of organic matters in the effluent of SBRs at different SRTs.

The concentration of the effluent SMP increased initially with increasing SRTs and then fell down. The effluent TOC of each SBR was 7.8 mg/L, 13.3 mg/L, 12 mg/L and 9 mg/L, respectively. As SMP was the product of sludge metabolism, less SMP produced at a low SRT of 5 d because of the low concentration of activated sludge. However, at the SRT of 40 d, more SMP might be produced, but the long SRT might also promote its degradation, resulting in a low concentration of the effluent SMP. UVA_254_ can be used to represent the humic content in the effluent. A small value of UVA_254_ at long SRTs illustrated that a high SRT might promote the degradation of humic substrate. Similar results were also obtained by Guo *et al.* [8]. Generally, MWD of dissolved organic matters in the effluent can be divided into three proportions: MW > 10,000 Da (10,000–30,000 Da), representing polysaccharides and proteins *etc.*; 500 Da < MW < 3,000 Da, representing the refractory organic matters, such as the humic substances; MW < 200 Da, representing the low molecular weight organic compounds, such as the glucose, acetate, and so on [37]. Figure 1 displays the typical chromatograms of effluents from reactors at different SRTs. Similar characteristics except for the intensity of the UV chromatograms were obtained. The UV chromatograms of the effluents had some sub-peaks in the range of 500–3,000 Da. Strong absorbance appeared at MW of 600 Da and 2,300 Da, demonstrating the presence of humic-like substances. These results were consistent with Jarustthirak and Amy [38] and Esparza-Soto *et al.* [39], where showed that SRT did not affect characteristics of the effluent MWD but the densities of the UV chromatograms were different.

### 3.2. Dynamics of Nutrients in SBR Cycles at Different SRTs

**Figure 2:**
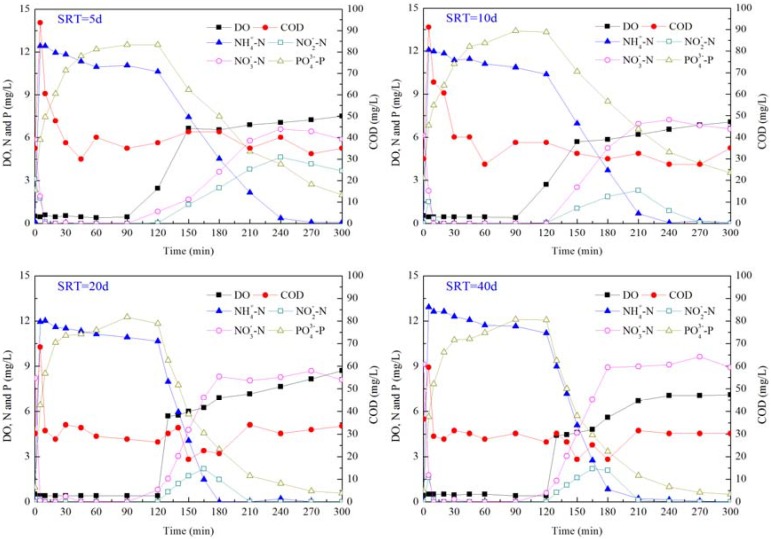
Dynamics of nutrients within a typical SBR cycle at different SRTs.

Profiles of NH_4_-N, NO_2_-N, NO_3_-N, PO_4_-P and COD in typical SBR cycles in all reactors are shown in Figure 2. In four SBRs, most of the COD were removed during the anoxic/anaerobic phase as the COD reached a concentration below 40 mg/L within 1 h after cycles started.

NO_3_-N and NO_2_-N were reduced quickly in the anoxic/anaerobic phase. In SBR5 and SBR10, denitrification was complete within 10 min, while in SBR20 and SBR40 denitrification was completed within 5 min, illustrating that denitrification was carried out efficiently in all reactors. NH_4_-N was removed slightly for cell synthesis in four reactors during the anoxic/anaerobic phase. The assimilated NH_4_-N in each reactor was 1.8 mg/L, 1.6 mg/L, 1.3 mg/L and 1.1 mg/L, respectively. A slightly high amount of NH_4_-N was assimilated at a low SRT, indicating a high growth rate of microorganisms under this condition. Most of NH_4_-N was removed in the aerobic phase by nitrification. In SBR5, NH_4_-N was completely removed at 2 h of the aerobic phase, at the same time, NO_2_-N reached the peak concentration and there was still a certain concentration of NO_2_-N at the end of the aerobic phase. In SBR10, SBR20 and SBR40, NH_4_-N was completely removed at 2 h, 1 h and 1.5 h, respectively. NO_2_-N was also accumulated in those three reactors, but with continuation of nitrification, NO_2_-N disappeared at 2.5 h, 1.5 h and 2 h of the aerobic phase in SBR10, SBR20 and SBR40, respectively. The volumetric nitrification rate of four reactors was 5.1 mg/(L·h), 6.5 mg/(L·h), 10.6 mg/(L·h) and 10.4 mg/(L·h). This showed that a long SRT could promote the nitrification efficiency and the optimal SRT for nitrification was 20 d.

EBPR is achieved with the enrichment of PAOs. PAOs take up carbon sources and hydrolyze polyphosphate in the anaerobic phase. During the aerobic phase, PAOs are able to accumulate phosphorus in excess, which is then removed through wasting residue sludge. Phosphorus release and uptake occurred in all reactors, indicating the presence of PAOs in these systems. During the anaerobic phase, the phosphorus release reached the peak value at the same time with the complete removal of COD. At SRTs of 5 d, 10 d, 20 d and 40 d, the released phosphorus was 7.4 mg/L, 7.5 mg/L, 8.5 mg/L and 8.6 mg/L, respectively, while the corresponding amount of phosphorus uptake was 10.5 mg/L, 9.8 mg/L, 11.2 mg/L and 11.6 mg/L. It was reported that the phosphorus uptake in the aerobic phase was about 1.3 times the phosphorus released in the anaerobic phase [36]. Ratios between phosphorus uptake and phosphorus release in this study were 1.41, 1.30, 1.32 and 1.35. The specific mass phosphorus release rates were in the range of 1.7–6.7 mg P/g VSS, and these were much lower than the reported values between 8.4–22.6 mg P/g VSS from other lab-scale studies [40], showing that the bioactivity of PAOs was relatively low in the present study.

### 3.3. Sludge Characteristics at Different SRTs

In an activated sludge process, an appropriate concentration of activated sludge is an important factor ensuring efficient nutrient removal. Concentrations of activated sludge in systems at different SRTs are shown in Table 2. With the SRT increased from 5 d to 40 d, the concentration of SS rose from 1,103 mg/L to 5,127 mg/L. The ratio between VSS and SS decreased with increasing SRTs. The VSS/SS ratios were 0.94, 0.91, 0.84 and 0.76 in SBR5, SBR10, SBR20 and SBR40, respectively. For a biological wastewater treatment system, the sludge concentration in the bioreactor is the net balance between microbial growth and endogenous respiration. The relationship between the observed sludge yield (*Y*_obs_) and the SRT can be described by the following equation [41]:

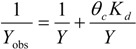
(1)
where *Y* is the true growth yield, g VSS/g COD; *θ*_c_ is the SRT, d; and *K*_d_ is specific endogenous rate, d^−1^. According to the daily influent and effluent COD and VSS, values of *Y*_obs_ were 0.46 g VSS/g COD, 0.34 g VSS/g COD, 0.29 g VSS/g COD and 0.26 g VSS/g COD, where *Y*_obs_ decreased with increasing SRTs. The true growth yield is not affected by the SRT. By substituting SRTs and *Y*_obs_ under different SRTs into the equation, the *Y* and *K*_d_ were obtained. The value of *Y* was 0.49 and that of *K*_d_ was 0.020 d^−1^, 0.036 d^−1^, 0.037 d^−1^ and 0.039 d^−1^ at the SRT of 5 d, 10 d, 20 d and 40 d, respectively. These results were similar to those of Al-Malack [42], where the yield (*Y*) and the endogenous decay coefficient (*K*_d_) were in the range of 0.487–0.583 mg VSS/mg COD and 0.0261–0.151 d^−1^, respectively.

SVI in the four SBRs ranged in 49–108 mL/g. Generally, SVI was low at low SRTs. The SVI was 49 mL/g and 23 mL/g at the SRT of 5 d and 10 d, respectively, whereas the SVI was 69 mL/g and 108 mL/g at the SRT of 20 d and 40 d, respectively. The production of EPS decreased with increasing SRTs. At a low SRT, with a high food to microorganism (F/M) ratio, excess carbon substrates might be converted to intracellular storage granules and EPS. At high SRTs, the production of EPS decreased due to a low F/M ratio. Protein was the dominant component in EPS, followed by carbohydrate. The concentration of carbohydrate was obviously small at high SRTs, and with the SRT increased from 5 d to 40 d, the concentration of carbohydrate decreased from 74.3 mg/L to 33.9 mg/L. However, the protein concentration only reduced slightly with increasing SRTs, in a range of 82.3–60.7 mg/L. The ratio of protein to carbohydrate increased from 1.11 at the SRT of 5 d to 1.93 at the SRT of 20 d and then decreased to 1.79 at the SRT of 40 d. Similar results were also obtained by Liao *et al.* [13], where the ratio of protein to carbohydrate increased with an increase of SRT from 4 d to 12 d and then remained constant with SRTs above 12 d. Statistical analysis was conducted to find the correlation among the total EPS, the ratio of protein to carbohydrate and the SVI. Results showed that the EPS production had a slightly positive effect on the SVI (R^2^ = 0.49, confidence level of 95%), but there seemed no obvious correlation between the protein to carbohydrate ratio and SVI (R^2^ = 0.31, confidence level of 95%).

**Table 2:** Sludge characteristics under different SRTs.

Parameter		Unit	Sludge Retention Time			
SRT		d	5	10	20	40
SS		mg/L	1,103 ± 80	1,449 ± 67	3,165 ± 130	5,127 ± 600
VSS		mg/L	1,041 ± 100	1,324 ± 104	2,682 ± 170	3,900 ± 350
SVI		mL/g	49 ± 18	23 ± 5	69 ± 5	108 ± 14
EPS	Total	mg/g VSS	157 ± 12	145 ± 19	111 ± 10	95 ± 10
	Protein	mg/g VSS	82.3 ± 12.4	78.6 ± 10.8	73 ± 4.4	60.7 ± 6.3
	Carbohydrate	mg/g VSS	74.3 ± 7.5	66.3 ± 9.8	37.8 ± 6.2	33.9 ± 6.8
Nitrification rate	1st step	mg/(g VSS·h)	5.4	3.9	3	2.4
	2nd step	mg/(g VSS·h)	1.3	2.4	1.4	1.3
Denitrification rate	1st step	mg/(g VSS·h)	15.6	10.6	13.8	9.4
	2nd step	mg/(g VSS·h)	7.6	6.5	8.8	2.9

Oxidation rates of nitrite at different SRTs were lower than those of ammonia, which explained the accumulation of nitrite in reactors during the aerobic phase. Moreover, reduction rates of nitrite at different SRTs were lower than those of nitrate, and this might be the main reason of the appearance of nitrite at the initial anoxic phase in all reactors. As shown above, the highest nitrite concentration in the aerobic phase occurred at the SRT of 5 d. Consistent with this, the lowest nitrite reduction rate and the highest ammonia oxidation rate were both obtained at the SRT of 5 d, and the difference between these reaction rates led to the nitrite accumulation. Although, at the SRT of 40 d, the mass nitrification rate and the mass denitrification rate (with the unit of mg N/(g VSS·h)) were relative low due to the high endogenous decay rate, but the highest volumetric reduction rate (with the unit of mg N/(L·h)) of NH_4_-N was obtained in SBR40. This was due to that SS concentration was high in SBR40 and a large amount of functional bacteria contained in this system, leading to a high volumetric reaction rate. Therefore, a balance between the mass activity and the net amount of activated sludge should be considered to achieve the optimal nutrient removal performance. As autotrophic nitrifiers grow slowly compared with other heterotrophs, the volumetric nitrification rate rose steadily with an increase in SRT. However, the volumetric denitrification rate increased with the SRT increased from 5 d to 20 d and then decreased thereafter. Therefore, to achieve the optimal nitrogen removal, the SRT should be controlled around 20 d.

### 3.4. N_2_O Emission in SBR Cycles at Different SRTs

**Figure 3:**
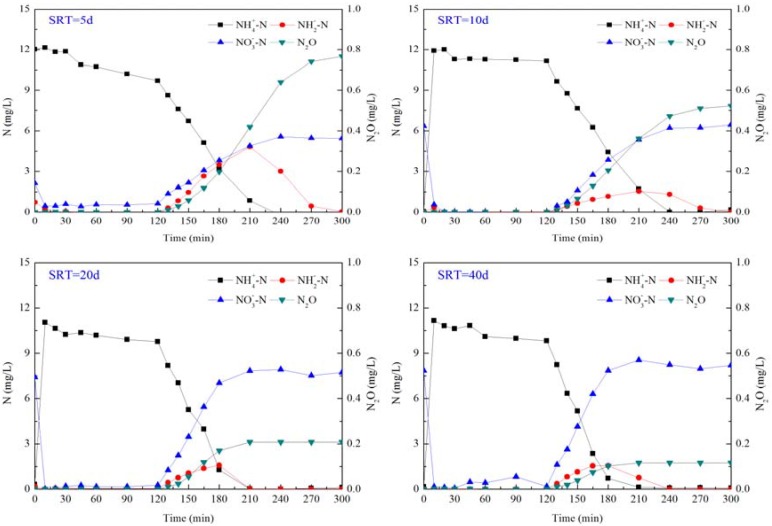
Dynamics of nitrogen in typical batch SBR cycles at different SRTs.

Batch reactors were used to investigate characteristics of N_2_O emission in cycles at different SRTs. Dynamics of NH_4_-N, NO_2_-N, NO_3_-N and N_2_O in batch SBR cycles are shown in Figure 3.

NO_3_-N was denitrified quickly in the anoxic/anaerobic phase in all reactors, and the N_2_O emission from denitrification was negligible. At SRTs of 5 d and 10 d, the N_2_O emission during the anoxic/anaerobic phase was 0.007 mg/L and 0.004 mg/L, respectively, accounting for 1‰ and 0.5‰ of the denitrified NO_3_-N. While at SRTs of 20 d and 40 d, the N_2_O emission during the anoxic/anaerobic phase were both below 0.002 mg/L, and accounted for less than 0.2‰ of the denitrified NO_3_-N. At all SRTs, the N_2_O emission from the aerobic phase were significantly higher than that from the anoxic/anaerobic phase, and similar phenomenon had been also reported by several other studies [25,43]. Therefore, the N_2_O emission during the nitrogen removal should focus on the aerobic phase. In the aerobic phase, the removed NH_4_-N was 9.7 mg/L, 11 mg/L, 9.7 mg/L and 8.5 mg/L, and the N_2_O emission was 0.49 mg/L, 0.33 mg/L, 0.13 mg/L and 0.07 mg/L. Ratios of N_2_O-N to the removed NH_4_-N in the aerobic phase were 5%, 3%, 1.3% and 0.8% under SRTs of 5–20 d. The emission ratio decreased with increasing SRTs, and this result was consistent with that of Noda *et al.* [25]. It had been clarified that N_2_O was an intermediate of incomplete nitrification and denitrification, and the accumulation of NO_2_-N was recognized as the main factor promoting the N_2_O emission [44]. In this study, the accumulation of nitrite was observed in all reactors. During the aerobic phase, the maximum accumulated nitrite was 4.8 mg/L at the SRT of 5 d, 1.6 mg/L at the SRT of 10 d and about 1.5 mg/L at both SRTs of 20 d and 40 d. A high concentration of NO_2_-N might be the main reason for the increased N_2_O emission at low SRTs. At SRTs of 20 d and 40 d, NH_4_-N and NO_2_-N were totally removed at min 210 of the cycle, and at the same time, N_2_O emission reached the highest value. However, at SRTs of 5 d and 10 d, NH_4_-N was totally removed at min 240 of the cycle, while NO_2_-N was still remained and N_2_O emission kept increasing. This phenomenon illustrated that, at SRTs of 5 d and 10 d, nitrifiers or heterotrophs would denitrify with NO_2_-N as the electron acceptor and release N_2_O after NH_4_-N had been removed completely. As the enzyme activity of heterotrophs for N_2_O reduction could be strictly inhibited at high DO concentrations [45], and N_2_O emission during this stage should be mainly from the nitrifier denitrification rather than from heterotrophic denitrification.

### 3.5. N_2_O Emission in Batch Experiments under Different Conditions

**Figure 4:**
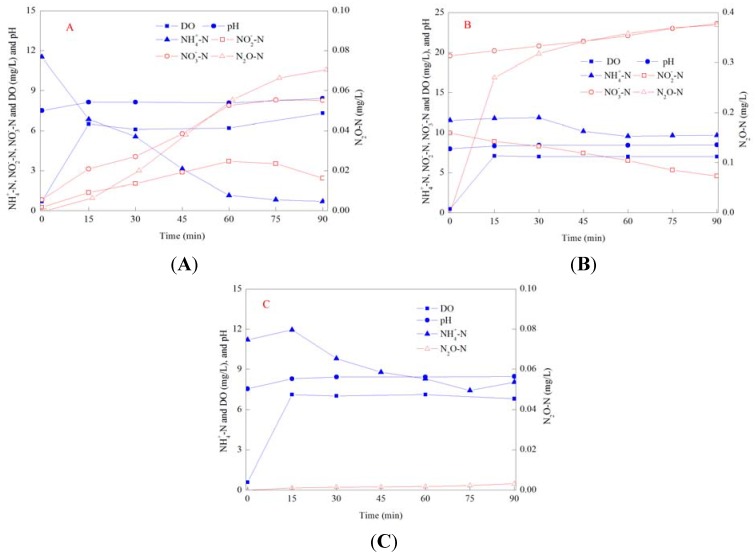
Batch experiments of N_2_O emission under different applied conditions. (**A**): without the addition of ATU and NOx-N; (**B**): with the addition of both ATU and NOx-N; (**C**): with the addition of ATU only.

N_2_O emissions in the SBR cycles showed that almost all of the N_2_O came from the aerobic phase but it was difficult to clarify the emission sources. Further batch experiments were carried out under the aerobic phase and the results are shown in Figure 4 (taken the SRT of 5 d as an example).

Under the conditions without ATU and oxidized nitrogen, characteristics of the N_2_O emission in batch experiments were the same as those in the aerobic phase in SBR cycles. With the removal of NH_4_-N, N_2_O emission increased and the highest accumulated concentration was 0.07 mg/L. Compared with the emission in SBR cycles, the N_2_O emission was relatively small in batch experiments. The reason was that the volume of the batch reactor was relative small and the oxygen transfer efficiency was high in a small reactor at the same oxygen loading rate. The maximum concentration of the accumulated NO_2_-N in batch experiments was also lower than that in the SBR cycle. The maximum accumulated nitrite was 3.8 mg/L in the batch experiment while the maximum accumulation was 4.8 mg/L in the SBR cycle.

Under the conditions with the addition of ATU and oxidized nitrogen, the N_2_O emission was 0.38 mg/L, which was much higher than that under the condition without ATU and oxidized nitrogen, illustrating that denitrification under the aerobic phase contributed significantly to the N_2_O emission. As nitrification was inhibited by ATU, there was no N_2_O produced from nitrification and all of the N_2_O emission should come from heterotrophic denitrification and/or nitrifier denitrification. The N_2_O emission could be divided into two different phases, and in the initial 10 min, the N_2_O emission rate was obviously higher than the emission rate in the following phase. The same phenomenon was obtained in the experiments under other three SRTs. But in the SBR cycles and under the condition without ATU, there was not an increased N_2_O emission rate in the initial aerobic phase. Therefore, the increased N_2_O emission was not caused by the air stripping and it was more likely owing to biological activities. At the end of the anoxic/anaerobic phase, there might be some residual carbon source in the batch reactor. In addition, a relative low DO concentration existed at the initial aerobic phase, due to the limitation of oxygen transfer efficiency. The low DO circumstance and the addition of oxidized nitrogen provided favorable conditions for heterotrophic denitrification, resulting in the promoted N_2_O emission. With aeration continued, DO reached a concentration of 6 mg/L, and this high DO concentration could restrict heterotrophic denitrification. Therefore, the following N_2_O emission should come from nitrifier denitrification.

At the initial aerobic phase of SBR cycles and the experiment under the condition without the addition of ATU and oxidized nitrogen, there might also be some residual carbon source and the low DO concentration, but the absence of oxidized nitrogen made heterotrophic denitrification impossible. Under the condition with ATU and oxidized nitrogen, the emission rate in the second phase of the experiment was much higher than that under the condition without the addition of ATU and oxidized nitrogen, which was mainly due to the added high concentration of oxidized nitrogen. Thus, effects of DO and oxidized nitrogen on nitrification and denitrification should be considered comprehensively to control the N_2_O emission during the aerobic phase and further studies are required.

## 4. Conclusions

The performance of COD removal and TP removal were similar under SRTs of 5–40 d; SRT mainly affected the nitrogen removal and the optimal SRT for BNR was 20 d. The MWD was in the range of 500–3,000 Da under SRTs of 5–40 d. The minimum effluent concentration of SMP was obtained at the SRT of 5 d.

The growth of nitrifiers was restricted at a short SRT and nitrite existed in the effluent of SBR5.

SS concentration increased with increasing SRTs while the excess sludge production reduced due to the increased endogenous decay at high SRT_S_. The endogenous decay coefficient was 0.020 d^−1^, 0.036 d^−1^, 0.037 d^−1^ and 0.039 d^−1^ at SRTs of 5 d, 10 d, 20 d and 40 d, respectively.

In BNR processes, the N_2_O emission was produced mainly during the aerobic phase and decreased with increasing SRTs. The ratio of N_2_O-N to the removed NH_4_-N in the aerobic phase was 5%, 3%, 1.8% and 0.8% at SRTs of 5 d, 10 d, 20 d and 40 d, respectively. With low DO concentrations and high oxidized nitrogen concentrations, the N_2_O emission was significantly accelerated by heterotrophic denitrification.
